# The longer, the better? Investigating the effect of prolonged acoustic stimulation on brief acoustic tinnitus suppression

**DOI:** 10.1186/s12883-026-04997-0

**Published:** 2026-06-30

**Authors:** Johanna Rischer, Patrick Neff, Berthold Langguth, Milena Engelke, Andreas Reissmann, Stefan Schoisswohl

**Affiliations:** 1 Department of Psychiatry and Psychotherapy, University of Regensburg, Regensburg, Germany; 2 Faculty of Medicine, University of Zurich, Zurich, Switzerland

**Keywords:** Tinnitus, Acoustic stimulation, Residual inhibition, Brief acoustic tinnitus suppression

## Abstract

**Background:**

Brief acoustic tinnitus suppression following sound stimulation is being studied to better understand the mechanisms underlying tinnitus and short-term suppression of the tinnitus perception. In this context, several kinds of filtered or modulated stimuli have been investigated. However, little research was conducted regarding the effect of stimulation length for brief acoustic tinnitus suppression.

**Methods:**

The aim of the present study was to compare the extent of tinnitus suppression after a 20-minute and a 3-minute acoustic stimulation with an individual best stimulus. Three experimental sessions were completed by 33 participants with chronic subjective tinnitus. In the first two sessions, eight different individualized, filtered, and/or amplitude-modulated stimuli were presented for 3 min each. For each participant, the stimulus which induced the strongest tinnitus loudness suppression (measured with a numeric rating scale in percent compared to baseline loudness) was chosen and then applied for 20 min in a third session.

**Results:**

On a group level, no significant difference in tinnitus loudness suppression comparing the 3-minute and the 20-minute acoustic stimulation using the individual best stimulus was observed. However, individual response patterns revealed great diversity as 12 participants showed better suppression after the 20-minute stimulation,12 experienced worse tinnitus suppression and for 9 participants no distinct change was evident compared to 3-minute acoustic stimulation.

**Conclusions:**

Future research should try to characterize the subgroup of tinnitus patients that profits from prolonged acoustic stimulation and search for optimized simulation durations.

**Trial registration:**

This trial was retrospectively registered on 2026/03/04 at ClinicalTrials.gov (ID: NCT07472257).

**Supplementary Information:**

The online version contains supplementary material available at 10.1186/s12883-026-04997-0.

## Introduction

The conscious perception of a sound or noise, in the absence of a corresponding external acoustic source is described as tinnitus. If this phantom auditory perception is present over a period of 3 months, it is defined as chronic [1, 2]. The international prevalence for tinnitus is 14.4%. Hence, it is estimated to affect more than 740 million adults globally, of whom about 120 million are severely impacted [3]. The exact pathophysiological mechanisms of tinnitus and their diversity between individuals remain incompletely understood [4–6].

Following acoustic stimulation (AS) with various types of filtered or modulated sounds, 40–90% of tinnitus patients experience some sort of brief acoustic tinnitus suppression (BATS) [7–13]. This phenomenon is traditionally referred to as residual inhibition [14] and has the potential to provide essential insights into the basic mechanisms of tinnitus perception as well as tinnitus suppression. Research in this area may provide the basis for improving sound-based treatment strategies [15–17]. Presumably, BATS is mediated via feed-forward inhibition, but the exact underlying mechanisms and dynamics of BATS are still not completely understood [6].

Based on animal experiments, Galazyuk et al. showed that the activation of metabotropic glutamate receptors after AS reduces spontaneous neural activity in neurons of the inferior colliculus. They suggest that the phenomenon of BATS in humans may involve similar mechanisms [18, 19]. Other human neuroimaging studies revealed an association between BATS and higher-level auditory association regions as well as auditory networks [6, 20].

Several electroencephalography studies have demonstrated that during BATS a temporary inversion of tinnitus-associated spontaneous brain activity takes place [12, 21–23].

Since the first structured experiments on BATS through AS by Feldmann et al. [15], extensive research has been conducted in order to identify important variables like stimulus type or loudness, hoping for more pronounced and longer-lasting suppression effects [9, 24, 25]. Originally, white noise (WN) was used for AS, but soon, a variety of different noises, tones and modulated stimuli were investigated [16, 26, 27]. Schaette et al. [28] suggested stronger suppression effects when the individual tinnitus frequency (ITF) falls within the stimulated frequency range. Reavis et al. [8] expanded on this research and demonstrated superiority of amplitude-modulated (AM) sounds if their carrier frequencies were close to the ITF. Furthermore, sounds within the ITF at different AM rates were observed to produce particularly better suppression than ITF pure tones [29]. Narrow-band noise with a centre frequency covering the region of hearing loss appears to be superior to simple WN [30]. Besides the stimulus type, there are other factors influencing the quality and quantity of BATS as well. Fournier et al. [9] have shown the importance of stimulation intensity, defining the minimal residual inhibition level as a new factor. Furthermore, BATS susceptibility has a negative correlation with tinnitus chronicity in patients with normal hearing abilities [27].

To date, the role of stimulation duration and its effects on BATS remains largely unexplored. A systematic review from 2021 compared different experiments regarding the applied stimulation lengths and concluded that the duration of BATS increases with stimulus presentation time [11]. For example, Mahboubi et al. applied individualized sounds of 60-min length via a web-based system and reported an average suppression duration of 75 min (81% of participants reported BATS) [31]. Neff et al. [24] compared the relevance of stimulation length (3 min vs. 6 min) when applying 10 Hz AM stimuli with an ITF carrier sound. No statistically significant difference between 3- and 6-minute stimulation with respect to BATS depth and duration was evident. Terry et al. compared individually chosen stimuli of 10 s to 10 min duration. Only responders to a one-minute stimulation were included. They proposed that the suppression time correlates linearly with the logarithm of application time [32].

The aforementioned experiments by Neff et al. [24] and Terry et al. [32] represent two important studies which directly compared different stimulation lengths within the same experiment. To further investigate the consequences of stimulation length for BATS, we aimed to contribute to this knowledge gap by examining whether a 20-minute AS (= longstim) with an individually optimized stimulus evokes superior suppression effects compared to a 3-minute AS (= shortstim).

## Methods

All participants were informed about the entire study procedure, possible side effects and benefits. They were given the opportunity to ask questions at any time. Written informed consent was collected before the start of the study. The experiment was approved by the ethics committee of the University of Regensburg, Germany (ethical approval number: 17–819 −101) and registered at ClinicalTrials.gov (ID: NCT07472257). There was no financial compensation for participation. This work represents a sub-experiment of a larger study. Other aspects of this study have already been published [12, 23, 33]. Participants included in the present analysis had to meet the following criteria: (i) experiencing BATS in at least one stimulus during session 1 or 2 and (ii) complete all three experimental sessions (see section *Experimental procedure and acoustic stimulation*).

### Participants

Tinnitus patients were recruited through the Interdisciplinary Tinnitus Centre in Regensburg, Germany. Eligible for participation were patients with chronic subjective tinnitus of at least six months duration and an age between 18 and 75 years. The cognitive and linguistic ability to understand and participate in the experiment and provide written informed consent was mandatory. Exclusion criteria were objective tinnitus or a tinnitus frequency below 1 kHz, hyperacusis, oropharyngeal infection and medication with psychoactive substances. None of the following should have applied within 3 months before the beginning of the experiment: start of any other concurrent tinnitus treatment or tinnitus-related studies, substance or alcohol abuse. In case of any treatable or identifiable causes of tinnitus (Meniere’s disease, otosclerotic changes, etc.) or any present psychiatric, neurological, or internal disease, study participation was not permitted.

### Psychometry

Before the start of the experiment, all participants were requested to fill out the German versions of the following questionnaires using SoSci Survey [34]: the Tinnitus Sample Case History Questionnaire (TSCHQ) [35], the Tinnitus Handicap Inventory (THI) [36, 37], the Tinnitus Questionnaire (TQ) [38, 39] and the Questionnaire on Hypersensitivity to Sound (GUF) [40].

### Experimental procedure and acoustic stimulation

The study at hand consisted of three separate experimental AS sessions. As part of the first session, audiometric and tinnitometric measurements were conducted (see sections *Audiometry* and *Tinnitometry* below). These were followed by AS with three different noise stimuli: WN, WN with a bandstop filter (WN_BS) and WN with a bandpass filter (WN_BP). Both filters were implemented with a bandwith of one octave around the ITF [41].

Session two took place 3 to 14 days after the first one. Hereby, five different pure tone stimuli were presented: one tone at the ITF, two at the ITF with amplitude modulation of either 10 or 23 Hz (AM_10Hz, AM_23Hz) and another two low-frequency tones (three octaves below the patients` ITF) with an amplitude modulation of 10 or 23 Hz as well (AM_10Hz_deep, AM_23Hz_deep). The selection of stimuli was based on previous research, showing promising results for different modulated noises and pure tones [8, 24, 25]. WN was implemented to enable comparability of the modulated sounds to a “baseline”, which has been investigated in many other experiments [8, 11, 42]. The deep versions were implemented to explore new stimuli for this experiment and to compare the effect of AM sounds regarding their carrier frequency [8, 11, 24, 25]. AS was conducted with Psychophysics Toolbox Version 3 [43] in Matlab (Matlab R2017a; Mathworks, USA). Also, stimuli were created in Matlab and underwent root-mean-square correction to balance levels between different stimuli. They were presented diotically with a 1000 ms fade-in and fade-out phase in randomized order (double-blinded) for 3 min within each session. As BATS is a phenomenon lasting from seconds to minutes, a short break of 6 min took place between each stimulus presentation in session one and two to ensure consistency between the stimulation rounds.

The third session was offered to patients experiencing BATS during session 1 or 2. In this session, the stimulus that induced the strongest BATS in session 1 or 2 was chosen and applied within the course of a 20-minute AS. To ensure comparability with other tinnitus research, this experiment also applied a shortstim duration of 3 min [8, 11, 25]. Since Neff et al. [24] showed no difference between an AS of 3 versus 6 min, the longstim interval in this experiment was expanded to 20 min in combination with personalizing individual stimuli, to explore if this would introduce a different effect. Tinnitus matching, tinnitus sensation level and minimum masking level were determined in all three sessions (please see section *Tinnitometry* for a description). For each presentation, stimulus loudness was calculated by sensation level plus 65 dB SPL with an upper limit of 85 dB SPL. All reported loudness measurements in this experiment refer to dB SPL if not explicitly stated differently.

**Fig. 1:**
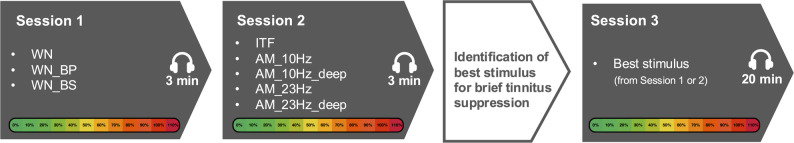
Acoustic stimulation procedure. This experiment consisted of three sessions including several acoustic stimulations. In session 1 three different noise stimuli were presented: White Noise (WN), WN with a bandpass filter (WN_BP) and WN with a bandstop filter (WN_BS), both filters applied at the individual tinnitus frequency (ITF). Stimuli presented in session 2 were five different pure tones: one at the ITF, two at the ITF with amplitude modulation at either 10 or –23 Hz (AM_10Hz, AM_23Hz) and another two low-frequency tones (three octaves below the patient`s ITF) with an amplitude modulation at 10 or –23 Hz as well (AM_10Hz_deep, AM_23Hz_deep). For each participant, the stimulus with best mean suppression during session 1 and 2 was chosen for the prolonged acoustic stimulation of 20 min as part of session 3. All stimuli were presented in a randomized order and double blinded within each session at a loudness of sensation level + 65 dB SPL with an upper limit of 85 dB SPL. Tinnitus loudness rating took place at seven points in time (every 30 s) following each stimulus application (t0, t30, t60, t90, t120, t150, t180) on a numeric rating scale anchored from 0–110%. 100% = tinnitus loudness is equal to pre-stimulation level, 110% = tinnitus loudness increased, e.g. 60% = present loudness is 60% of original tinnitus loudness. (Reference headphone icon: Freepik Company S.L. Headphones-Icon [Internet]. 2010–2025 [cited 2020 Feb 2]. Available from: https://www.flaticon.com/de/kostenloses-icon/kopfhorer_772183?term=kopfh%C3%B6rer&page=1&position=10&origin=tag&related_id=772183

Right after the end of each AS, participants had to rate the loudness of their tinnitus percept on a numeric rating scale in percent from 0 to 110 (explanation of the scale: 110% = 10% louder than before, 100% = same volume as before, 70% = only 70% of the original volume, 0% = total absence of tinnitus percept) [23]. This rating took place at seven time points: t0, t30, t60, t90, t120, t150 and t180 (t = seconds after AS end). For the longstim in session 3, ratings at time points t210, t240, t270, t300, t330, t360, t390, t420, t450, t480, t510, t540, t570 and t600 were added as longer periods of tinnitus suppression were hypothesized. Post-stimulation tinnitus loudness rating was performed on a customized keyboard strip (X-Key-Stick-16-USB, XK-0981-UCK16-R; P.I. Engineering, USA). For an illustration of the study procedure see Fig. 1.

### Audiometry

Software used for audiometry was the toolbox MultiTreshold (University of Essex, United Kingdom) using the implemented paradigm absolute threshold (absTreshold, an application of the two-alternatives forced-choice threshold estimation algorithm by *Green 1993 *[44]) in Matlab (Matlab R2017a; Mathworks, USA). To examine participants` hearing thresholds, each side was tested separately with sine tones (0.5 s) including frequencies from 250 Hz to 16 kHz on an octave scale. Presented loudness was 30 dB at the beginning and was increased in 10 dB steps, until the participants were able to hear the sound. Between trials, loudness level was increased or decreased in 2 dB steps, respectively. For tinnitus matching, AS, determination of participants` hearing thresholds, as well as sensation level and minimum masking level determination, ER-2 Tubephone insert earphones were used (Etymotic Research, Inc., Illinois), along with an external soundcard (RME Fireface UCX; Audio AG, Germany).

### Tinnitometry

Tinnitus matching was performed with a custom software tool (MAX 7; Cycling’74, USA) and a procedure of adjustment approach was chosen [30, 45]. Hardware for the matching was a custom-built controller including a Teensy 3.2 USB-based microcontroller (PJRC, USA), industrial-grade rotating knobs, switches and motor faders. Participants were schooled to match frequency and volume themselves through a matching device (starting loudness: participants` individual hearing threshold + 10 dB, starting frequency: one frequency group lower than the frequency with the greatest hearing loss, general frequency ranged from 40 to 16000 Hz), an octave confusion test followed afterwards. Also lateralization of the tinnitus sensation was adjusted through a fader by participants. If patients experienced more than one pitch within their tinnitus, they were instructed to match their most dominant pitch. Eventually, tinnitus consistency was rated on a 10-point Likert scale (1 = no resemblance, 10 = perfect resemblance). This procedure was repeated four times; the best rating represented the ITF and was chosen for creation of the individual stimuli used for AS (or the frequency closest to the mean frequency in case of similar ratings). Subsequently, minimum masking level was determined by increasing the intensity of a WN until complete tinnitus masking. Sennheiser HDA 2000 headphones (Sennheiser, Germany) were utilized to detect the loudness discomfort level in relation to participants` ITF, using the discomfort paradigm of the MultiTreshold toolbox. A detailed description of the matching procedure can be found in Neff et al. [29].

### Statistical analysis

Statistical analysis was carried out using R (R Foundation for Statistical Computing, Vienna, Austria, Version 4.2.3., packages: “lme4”, “lmerTest”, “psych”, “sjstats”, “emmeans” and “ggplot2”). In order to evaluate the effect of stimulation duration on BATS, a linear mixed effect model was defined using the following specifications: “subject” was classified as a random effect, whereas the variables *condition* (longstim, shortstim)”, rating *time* (t0, t30, t60, t90, t120, t150, t180 seconds) and interaction *condition x rating time* were classified as fixed effects. Tinnitus loudness (%) was determined as the dependent variable. The following model was tested for significant fixed effects via expected mean square approach: tinnitus loudness ~ condition + rating time + condition*rating time + (1|subject)). Significant fixed effects were then evaluated with post hoc Tukey tests and adjusted for multiple comparisons. The significance level was set at 5%.

To capture differences between shortstim and longstim beyond the group-level, as well as variability in temporal BATS dynamics, we descriptively compared tinnitus loudness ratings at every time point (t0-t180) at the individual subject level. For each participant and rating time (e.g., t0 longstim vs. t0 shortstim), we evaluated whether tinnitus suppression was superior in the longstim condition (yes/no). Participants were then classified into the following groups: *better tinnitus suppression* was defined as a lower tinnitus loudness rating in at least 4 out of 7 time points in the longstim condition, whereas *worse tinnitus suppression* was defined as a higher tinnitus loudness rating in at least 4 out of 7 time points. If none of these applied, participants were classified as *unchanged tinnitus suppression*.

## Results

Out of a total number of 45 patients, 33 (11 female) participated in all three experimental sessions. They exhibited BATS following one of the eight used stimuli during session 1 or 2 (shortstim), which was then applied in a prolonged AS of 20 min (longstim) in session 3. These participants were thus included in the present analyses.

### Descriptive data

Tinnitus was perceived in both ears by 22 patients, in the right ear by eight and in the left ear by three participants. Mean age was 51.88 years (SD = 12.68), with an average tinnitus duration of 90.27 months (SD = 59.81). Mean psychometry scores showed a moderate severity level according to the TQ (M = 39.21, SD = 14.43), a mild handicap in the THI (M = 34.36, SD = 19.42) and a moderate hypersensitivity to sounds according to the GUF score (M = 10.30, SD = 6.47). Detailed sample characteristics can be found in Table 1.

**Table 1:** Sample characteristics

	*N*	M	SD	Md	Min.	Max.
*N* (female)	33 (11)					
Tinnitus side (left/right/both)	3/8/22					
Age (years)	33	51.88	12.68	55.00	23.00	67.00
Duration (months)	33	90.27	59.81	72.00	18.00	224.00
TQ Score (0–84)	33	39.21	14.43	37.00	17.00	69.00
THI Score (0–100)	33	34.36	19.42	34.00	4.00	80.00
GUF Score (0–45)	33	10.30	6.47	10.00	0.00	23.00
Tinnitus frequency (Hz)	33	6071.03	2927.40	5900.00	1002.00	15424.00
Tinnitus loudness (dB)	33	51.67	13.83	49.00	27.00	78.00
SL (dB)	33	47.64	15.32	49.00	21.00	73.00
MML (dB)	33	61.03	14.35	58.00	33.00	90.00
HL left (dB)	33	22.60	13.35	24.58	−4.20	56.63
HL right (dB)	33	22.97	11.33	23.88	−3.89	48.40
HL total	33	22.78	11.84	23.76	−2.61	46.65
LDL left (dB)	18	86.33	3.01	85.50	81.00	90.00
LDL right (dB)	15	85.20	4.06	87.00	78.00	90.00

Tinnitus frequency, tinnitus loudness, SL and MML were evaluated in the third session

*N* number of participants, *M* mean, *SD* standard deviation, *Md* median, *Min.* minimum, *Max.* maximum, *TQ* Tinnitus Questionnaire, *THI* Tinnitus Handicap Inventory, *GUF* German Questionnaire on Hypersensitivity to Sound, *SL* sensation level, *MML* minimum masking level, *HL* hearing loss, *LDL* loudness discomfort level

### Statistical results

**Fig. 2:**
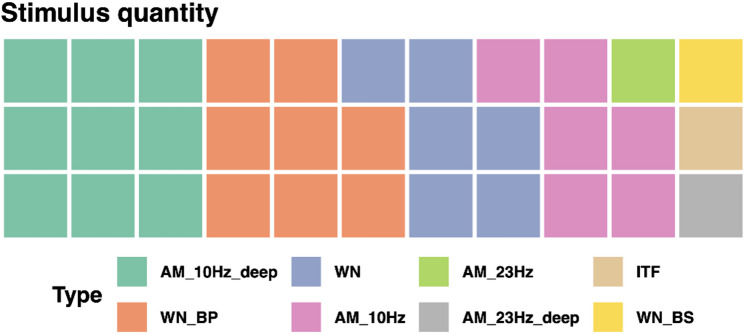
Individual best stimuli used for prolonged acoustic stimulation presented as a waffle chart. Each square represents one participant. The color represents the individual best stimulus type for short-term tinnitus suppression, which was then chosen for a prolonged acoustic stimulation of 20 min for the respective patient. ITF = individual tinnitus frequency; AM_10Hz = pure tone at the ITF with 10 Hz amplitude modulation; AM_23Hz = pure tone at the ITF with 23 Hz amplitude modulation; AM_10Hz_deep = pure tone three octaves below the ITF with 10 Hz amplitude modulation; AM_23Hz_deep = pure tone three octaves below the ITF with 23 Hz amplitude modulation; WN = white noise; WN_BP = WN with a bandpass filter implemented one octave around the ITF, WN_BS = WN with a bandstop filter implemented one octave around the ITF

The individually most effective stimuli inducing BATS, which were subsequently used for the longstim, can be found in Fig. 2. From the eight tested stimuli in the first two study sessions, AM_10Hz_deep appeared to be the most effective stimulus for 27.3% of participants, followed by WN_BP for 24.2% of participants.

**Fig. 3:**
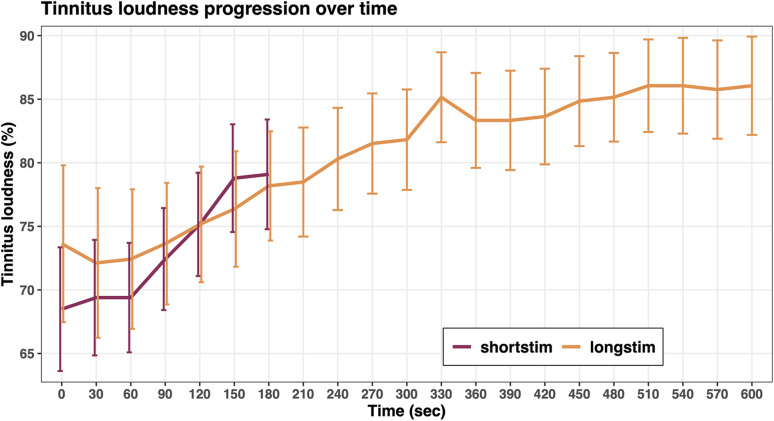
Tinnitus loudness progression over time. Mean tinnitus loudness for all rating time points for the conditions shortstim (purple) and longstim (orange). For longstim, the loudness progression is plotted up to 600 s following stimulation end. Error bars indicate 95% confidence intervals longstim = 20 min acoustic stimulation, shortstim = 3 min acoustic stimulation

Linear mixed effect model analysis revealed a significant effect of rating *time* (F = 2.152, *p* = 0.047) and no significant effect of *condition* or interaction of *condition x rating time* (*p* > 0.05) on tinnitus loudness. Subsequent post hoc tests for time failed to show significant differences between any time points. Loudness progression for shortstim and longstim is demonstrated in Fig. 3. Tinnitus loudness suppression was strongest directly after stimulation offset for both conditions, with a marginally higher suppression following the shortstim. Over time, tinnitus loudness increased more slowly after longstim than after the shortstim. However, these differences are not significant.

Detailed information on fixed effect testing results are provided in Table S1, descriptive statistics for loudness ratings per condition and time in Tables S2 - S4.

**Fig. 4:**
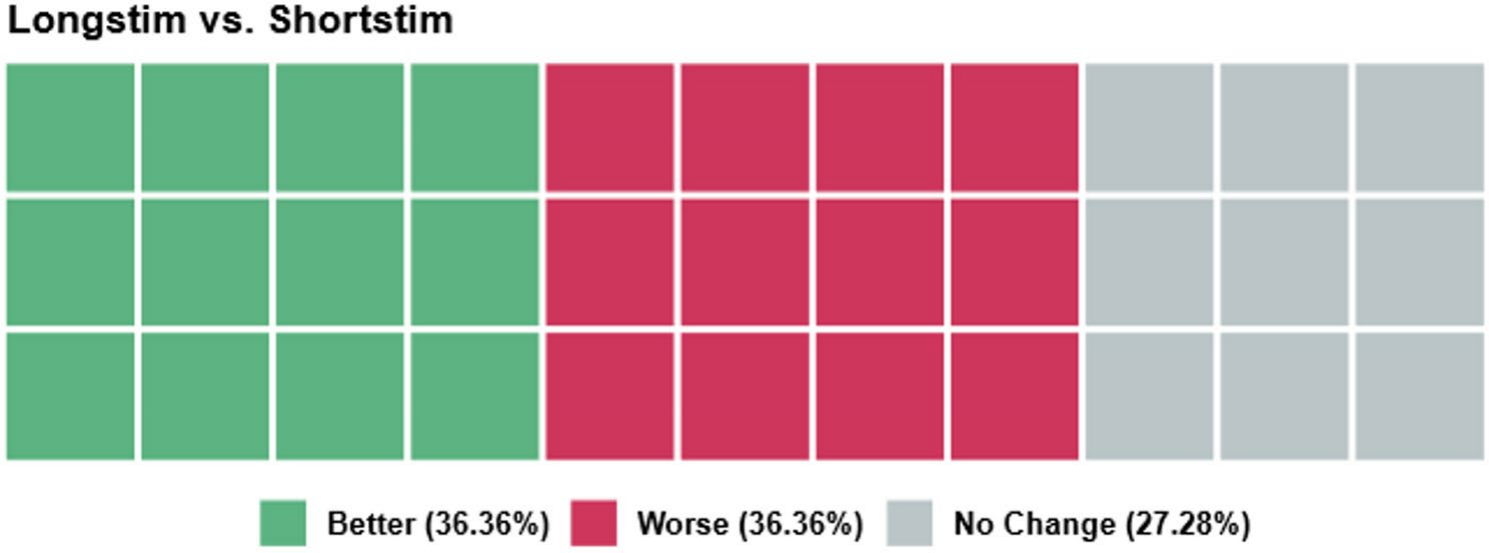
Individual comparison of longstim vs. shortstim response. Comment Each square represents one participant. Tinnitus loudness suppression was measured at 7 time points after acoustic stimulation on a numeric rating scale. If participants showed an improvement or a worsening in at least four out of seven ratings in longstim compared to shortstim, they were allocated to the categories better tinnitus suppression or worse tinnitus suppression respectively. Otherwise, they were classified to the category unchanged tinnitus suppression longstim = 20 min acoustic stimulation, shortstim = 3 min acoustic stimulation, Better = better tinnitus suppression for longstim vs. shortstim, Worse = worse tinnitus suppression for longstim vs. shortstim, No change = unchanged tinnitus suppression for longstim vs. shortstim

Based on the patient-specific descriptive comparison between longstim, shortstim and subject classification it can be reported that 12 participants (36.36%) showed better tinnitus suppression after prolonged AS using their individual best stimulus. For 9 participants (27.28%) longstim did not yield improvements. A further 12 participants (36.36%) exhibited worse tinnitus suppression. See Fig. 4 for an overview.

## Discussion

The present study compared two different AS lengths of 3 and 20 min with respect to BATS. For each participant, an individual best stimulus for BATS induction was chosen from a set of 8 different 3-minute sounds and applied in a prolonged AS of 20 min. With the present investigation we can put into perspective, that a prolonged AS with an individual best stimulus does not enhance BATS on a group level.

To the best of our knowledge, Terry et al. [32] were the first to investigate the influence of stimulus duration on participants, who experienced BATS at least once during different 1 min AS. In contrast, in the present study, sounds were presented for 3 min in order to identify the individual best AS stimulus. While Terry et al. compared AS durations of 10 s to 10 min with an applied stimulation loudness of minimum masking level + 20 dB, the present study compared 3 and 20 min of AS with an applied stimulation loudness of sensation level + 65 dB. The stimuli applied in the present experiment were AM and filtered sounds, respectively personalized according to the participants` tinnitus characteristics, whereas in the experiment of Terry et al., only narrow-band noises and pure tones at different preset frequencies were tested. Furthermore, information regarding tinnitus loudness rating intervals is missing. Altogether, they reported a logarithmic relation of stimulation duration with tinnitus suppression duration, while we did not observe any differences between 3- and 20-minutes AS on a group level.

Neff et al. [24] tested the effect of stimulation length by contrasting 10 Hz amplitude-modulated stimuli with an ITF carrier sound of 3- and 6-minute length presented at sensation level + 60 dB (= 5 dB difference to the present experiment). Tinnitus loudness rating time points and scales were equal to this experiment. All participants were included regardless of their initial response to AS and had to suffer from tinnitus for at least 12 months (instead of six months in the present experiment). No increase in BATS quality and length following a prolonged AS was present in their experiment.

Even though the duration of AS in our experiment was nearly tripled (20 min) and individual best stimuli for BATS were used, we did not observe any superiority of a prolonged AS as well.

Potentially, the longer application of an unpleasant sound may have produced some kind of aversive reaction and thus biased the tinnitus loudness rating afterwards, especially since stimuli were applied near or at the ITF. Also, some kind of saturation effect may be possible. Future studies could test more different stimulation lengths and may detect a time span, from which an increase in stimulation time will not be followed by an increase of BATS. As there is no standard approach in BATS research, various stimulus lengths and types were evaluated across different studies [24, 31, 32]. For example, Mahboubi et al. [31] applied an extensively prolonged AS of 60-minutes using narrow band noise filtered according to patient’s ITF and was able to demonstrate prolonged suppression effects exceeding the stimulation duration (75 ± 132 min). Without a direct comparison of stimulation lengths within the same experimental design, there is also no clear evidence of the most efficient stimulation time span, which impedes the collation of different experimental results.

In summary, prolonged stimulation did not improve the quality and duration of the BATS phenomenon in the present experiment. Nevertheless, enhanced suppression of the tinnitus percept may still occur at the individual level. A variation of individual response patterns is typically observed in experiments on BATS [9, 30, 31, 46]. In some cases, participants even experience longer-lasting suppressions of several hours or even days [16]. Hence, we investigated potential differences on an individual patient level based on predefined criteria (see section “Methods”) and could reveal heterogenous response patterns. With the present findings, it can be put into perspective that 12 patients reported an improvement in tinnitus suppression following 20-minof AS in contrast to a 3-min stimulation with an individual best stimulus. On the other hand, further 12 participants reported a worsening of BATS due to a 20-minute stimulation. Briefly, these results emphasize the need for further research investigating individual response patterns and the clinical implications behind this knowledge. Individualized stimuli based on a patient’s tinnitus suppression characteristics could be helpful in the future to maximize BATS, if a valid prediction of reactions on stimuli variables (e.g. stimulus type, stimulation loudness, stereo spectrum) can be determined and applied in clinical settings. Furthermore, research regarding the predictors, which individual participants might especially benefit from a prolonged stimulation could also improve the approach towards individualized treatment.

The results of previous studies suggest that stimuli close or within the ITF, as well as AM sounds induce superior BATS [8, 24, 29, 30, 42]. Interestingly, in 27% of subjects a 10 Hz AM tone with a carrier frequency of 3 octaves below the ITF appeared to be the most effective stimulus and was subsequently used for prolonged stimulation. A potential explanation for this finding could be, that such a deep sound was more comfortable to listen to and tinnitus loudness was still suppressed despite the low frequency of the carrier sound. In future studies the collection of valence and arousal ratings should be considered, especially in prolonged stimulation settings, to detect the possible aversive effects of a certain stimuli.

To the best of our knowledge, this is the first experiment to apply stimuli 3 octaves below patients ITF. Future experiments with other manipulated low frequency stimuli may provide further insights into the effects of carrier frequencies beyond those close to the ITF. In accordance with previous research [24, 29], our findings clearly support an amplitude modulation rate of 10 Hz, regardless of the octave manipulation as effective stimulus modulation. Furthermore, investigating the test-retest reliability of BATS for certain stimulus types could facilitate a more precise interpretation and exclude coincidental findings.

### Limitations

For the adequate interpretation of the results, some information should be considered: Group-based statistics are limited due to the sample size. As only participants showing BATS after shortstim were included, the investigated sample is not representative for all tinnitus patients. However, as the research question was if BATS could be optimized through a longer AS duration, pre-selection was mandatory. Post-stimulation evaluation of tinnitus loudness ended 180 s after stimulation end for shortstim, respectively 600 s after the end of longstim. A comparison between the effects of shortstim and longstim was only possible for 180 s after stimulation, so that potential differences beyond 180 s cannot be excluded. Also, the tinnitus loudness rating was a subjective measurement, as currently no objective methods exist and correlation of different methods are poor [47]. Moreover, AS itself may influence the rating behaviour of participants, if their memory of pre-stimulation loudness gets distorted, which influences their loudness assessment from pre- to post-stimulation ratings. In addition, other order effects within or between the sessions could have been introduced. Finally, this experiment was based on the hypothesis that BATS after shortstim is associated with BATS after longer stimulations, which may not be dependent at all.

## Conclusion

The current study investigated the effect of stimulus duration on brief acoustic tinnitus suppression. No significant difference between the two stimulation lengths (3 min vs. 20 min) was evident. The variety of responder profiles indicates the need for future research to better understand different response patterns after acoustic stimulation and their predictors, in order to individualize acoustic stimulation settings in the future.

## Supplementary Information

Supplementary Material 1.

## Data Availability

The datasets used and/or analyzed during the current study are available from the corresponding author on reasonable request.

## Abbreviations

AS: Acoustic stimulation
BATS: Brief acoustic tinnitus suppression
AM: Amplitude modulated
WN: White Noise
ITF: Individual tinnitus frequency
BP: Bandpass
BS: Bandstop
Longstim: Acoustic stimulation of 20 min duration
Shortstim: Acoustic stimulation of 3 min duration
