# Supplementary material for: The longer, the better? Investigating the effect of prolonged acoustic stimulation on brief acoustic tinnitus suppression
Source: BMC Neurol. 2026 Jun 30;26:417. doi: 10.1186/s12883-026-04997-0 (PMC13317285; doi:10.1186/s12883-026-04997-0)
Supplement: Supplementary file 1 — Supplementary Material 1. [file 12883_2026_4997_MOESM1_ESM.docx]

**Supplementary Material - The longer, the better? Investigating the effect of prolonged acoustic stimulation on brief acoustic tinnitus suppression**

**Table S1: Fixed effect testing results**

Tested Model: *Tinnitus loudness ~ condition + rating time + condition*rating time + (1|ID)*

|  | **numDF** | **denDF** | **F** | **p-value** |
| --- | --- | --- | --- | --- |
| Condition | 1 | 429 | 0.547 | 0.460 |
| **Rating time** | **6** | **429** | **2.152** | **0.047** |
| Condition*Rating time | 6 | 429 | 0.333 | 0.920 |

**Table S1: Fixed effect testing results** numDF, degrees of freedom numerator; denDF, degrees of freedom denominator

**Table S2: Descriptive results of tinnitus loudness ratings**

| **Rating time** |  | | | | |
| --- | --- | --- | --- | --- | --- |
|  | **M** | **SD** | **Md** | **Min** | **Max** |
| T0 | 71.06 | 31.77 | 80 | 0 | 110 |
| T30 | 70.76 | 29.99 | 80 | 0 | 110 |
| T60 | 70.91 | 28.16 | 80 | 0 | 110 |
| T90 | 73.03 | 25.17 | 80 | 10 | 110 |
| T120 | 75.15 | 24.57 | 80 | 10 | 110 |
| T150 | 77.58 | 25.06 | 80 | 0 | 110 |
| T180 | 78.64 | 24.55 | 90 | 0 | 110 |

**Table S2: Descriptive results of tinnitus loudness ratings** Tinnitus loudness was measured at seven time points after stimulation end in 30-second intervals. T0 represents the first tinnitus loudness rating directly after stimulation end and T”X” the time points of the other ratings in seconds. Tinnitus loudness was rated on a numeric rating scale ranging from 0 to 110% (110% - increase in tinnitus loudness, 100% - tinnitus loudness maintained its pre-stimulation levels, 50% - tinnitus loudness equals half of the pre-stimulation loudness). M = Mean, SD = Standard Deviation, Md = Median, Min = Minimum, Max = Maximum

**Table S3: Descriptive results of the variable “condition”**

| **Longstim** | | | | | **Shortstim** | | | | |
| --- | --- | --- | --- | --- | --- | --- | --- | --- | --- |
| **M** | **SD** | **Md** | **Min** | **Max** | **M** | **SD** | **Md** | **Min** | **Max** |
| 74.50 | 29.25 | 80 | 0 | 110 | 73.25 | 24.98 | 80 | 0 | 110 |

**Table S3: Descriptive results of the variable “condition”** Longstim = long stimulation (20-minute stimulation duration), Shortstim = short stimulation of (3-minute duration), M = mean, SD = standard deviation, Md = median, Min = minimum, Max = maximum

**Table S4: Descriptive results of interaction “condition × rating time”**

| **Rating Time** | **Longstim** | | | | | **Shortstim** | | | | |
| --- | --- | --- | --- | --- | --- | --- | --- | --- | --- | --- |
|  | **M** | **SD** | **Md** | **Min** | **Max** | **M** | **SD** | **Md** | **Min** | **Max** |
| T0 | 73.64 | 35.43 | 80 | 0 | 110 | 68.48 | 27.96 | 80 | 20 | 110 |
| T30 | 72.12 | 33.80 | 80 | 0 | 110 | 69.39 | 26.09 | 80 | 0 | 110 |
| T60 | 72.42 | 31.53 | 90 | 0 | 110 | 69.39 | 24.74 | 80 | 0 | 100 |
| T90 | 73.64 | 27.48 | 80 | 10 | 110 | 72.42 | 23.05 | 80 | 10 | 100 |
| T120 | 75.15 | 26.12 | 80 | 20 | 110 | 75.15 | 23.33 | 80 | 10 | 100 |
| T150 | 76.36 | 26.08 | 80 | 20 | 110 | 78.79 | 24.34 | 90 | 0 | 100 |
| T180 | 78.18 | 24.68 | 80 | 20 | 110 | 79.09 | 24.79 | 90 | 0 | 100 |

**Table S4: Descriptive results of variable “condition × rating time”** Longstim = long stimulation (20-minute stimulation duration), Shortstim = short stimulation of (3-minute duration), M = mean, SD = standard deviation, Md = median, Min = minimum, Max = maximum
